# Hesitancy to Undergo SARS-CoV-2 Rapid Antigen Testing in China: Nationwide Cross-sectional Study

**DOI:** 10.2196/43555

**Published:** 2023-05-01

**Authors:** Zhen Lu, Leiwen Fu, Luoyao Yang, Tian Tian, Yanxiao Gao, Xiaojun Meng, Huachun Zou

**Affiliations:** 1 School of Public Health (Shenzhen) Sun Yat-sen University Shenzhen China; 2 Wuxi Municipal Center for Disease Control and Prevention Wuxi China

**Keywords:** COVID-19, SARS-CoV-2, vaccine, hesitancy, rapid antigen testing, China

## Abstract

**Background:**

SARS-CoV-2 rapid antigen testing (RAT) could be a useful supplementary test to diagnose larger numbers of acute asymptomatic infections and alleviate the limitations of polymerase chain reaction testing. However, hesitancy to undergo SARS-CoV-2 RAT may compromise its implementation.

**Objective:**

We aimed to understand the prevalence and correlates of hesitancy to undergo RAT among adults not infected with SARS-CoV-2 in mainland China.

**Methods:**

A nationwide cross-sectional survey on hesitancy to undergo SARS-CoV-2 RAT was conducted among adults not infected with SARS-CoV-2 in mainland China between April 29, 2022, and May 10, 2022. Participants completed an online questionnaire that covered the following COVID-19–related factors: sociodemographic characteristics, experiences of COVID-19 restrictions and knowledge of COVID-19, and attitude toward COVID-19 and its screening. This study was a secondary analysis of data from the survey. We compared the characteristics of participants by hesitancy to undergo SARS-CoV-2 RAT. Thereafter, logistic regression with a sparse group minimax concave penalty was used to identify correlates of hesitancy to undergo RAT.

**Results:**

We recruited 8856 individuals with diverse demographic, socioeconomic, and geographic characteristics in China. Eventually, 5388 participants (valid response rate of 60.84%; 52.32% [2819/5388] women; median age 32 years) were included in the analysis. Among the 5388 participants, 687 (12.75%) expressed hesitancy to undergo RAT and 4701 (87.25%) were willing to undergo RAT. Notably, those who were from the central region (adjusted odds ratio [aOR] 1.815, 95% CI 1.441-2.278) and those who received COVID-19 information from traditional media (aOR 1.544, 95% CI 1.279-1.863) were significantly more likely to report hesitancy to undergo RAT (both *P*<.001). However, those who were women (aOR 0.720, 95% CI 0.599-0.864), were older (aOR 0.982, 95% CI 0.969-0.995), had postgraduate education (aOR 0.612, 95% CI 0.435-0.858), had children (<6 years old) and elders (>60 years old) in the family (aOR 0.685, 95% CI 0.510-0.911), had better knowledge about COVID-19 (aOR 0.942, 95% CI 0.916-0.970), and had mental health disorders (aOR 0.795, 95% CI 0.646-0.975) were less likely to report hesitancy to undergo RAT.

**Conclusions:**

Hesitancy to undergo SARS-CoV-2 RAT was low among individuals who were not yet infected with SARS-CoV-2. Efforts should be made to improve the awareness and acceptance of RAT among men, younger adults, individuals with a lower education or salary, families without children and elders, and individuals who access COVID-19 information via traditional media. In a reopening world, our study could inform the development of contextualized mass screening strategies in general and the scale-up of RAT in particular, which remains an indispensable option in emergency preparedness.

## Introduction

Multiple variants of SARS-CoV-2 have occurred [1], of which the Omicron variant has spread swiftly across the world and has become the progressive strain globally [2-5]. During the period of the COVID-19 pandemic, policy makers from various countries and regions have been focusing on efforts to mitigate the pandemic’s devastating impacts on public health, economy, and social development. COVID-19 vaccination and nonpharmaceutical interventions (NPIs) are expected to contain the pandemic to a large extent. Across the world, as of August 11, 2022, there were 34 COVID-19 vaccines that had been authorized for use [6]. In addition, many countries implemented NPIs, including face mask wearing, hand washing, and physical distancing, in response to the ongoing COVID-19 pandemic [7].

When the genetic sequence of SARS-CoV-2 was published in January 2020 [8], 3 types of diagnostic tests for COVID-19 were implemented, including polymerase chain reaction (PCR) testing, rapid antigen testing (RAT), and serology testing. While PCR testing and RAT could be used to diagnose an acute infection involving COVID-19, serology testing provides indirect evidence of infection 1-2 weeks after the onset of symptoms [9] and thus was not applied to large-scale screening in the early phase. PCR testing is highly sensitive and specific, and requires the support of laboratory facilities and highly trained staff. Its results become available in the time range of less than 2 hours to up to 7 days [10]. However, a delay of several days in receiving testing results is unacceptable for mass screening, as it may lead to missed diagnosis of many cases, which would cause secondary transmission. RAT, on the other hand, is a useful supplementary testing approach that detects viral proteins (eg, spike and nucleocapsid proteins) to diagnose larger numbers of acute asymptomatic SARS-CoV-2 infections, and it can overcome the limitations of PCR testing in the early phase of infection in mass screening. Of note, RAT requires minimal effort of medical personnel training and is much faster (within 15 minutes) at providing results [11,12]. Hence, RAT could confirm or exclude infection with SARS-CoV-2 at the individual level for case management or self-isolation, and at the population level for large-scale screening and emergency responses.

To tackle sporadic COVID-19 outbreaks, testing for large-scale screening should be one of the backbones of the response [13]. China has adopted the general strategy of “guarding against imported cases and preventing a resurgence of the outbreak at home” and the policy of “dynamic zero-COVID,” which include large-scale screening in a timely manner [14]. Furthermore, in March 2022, China implemented RAT to successfully identify infected individuals in the early phase in Shanghai. RAT has been used at universities and other congregate settings in the United States [12]. Germany, France, and the United Kingdom have already adopted RAT in their own strategies [15]. RAT is very useful in surveillance and emergency responses; however, the public may lack knowledge of RAT, that is, when and how to undergo RAT, or may be hesitant to undergo the procedure. In the case of outbreaks of COVID-19, especially those involving the Omicron variant, optimization of strategies of RAT for individuals in order to exert a dramatic effect on COVID-19 spread should be carefully considered. To address the transition from a pandemic to an endemic, evidence-based optimization of diagnostic strategies should be considered in the global agenda. Many studies have assessed reasons for COVID-19 vaccination hesitancy [16,17]; however, research characterizing hesitancy and the factors influencing hesitancy to undergo RAT for COVID-19 is scarce. Our study aimed to understand the prevalence and correlates of hesitancy to undergo RAT for COVID-19 among adults uninfected with SARS-CoV-2 in mainland China. For the COVID-19 pandemic and outbreaks of novel infectious diseases, the findings of this study could inform the future decision-making of the scale-up of RAT.

## Methods

### Study Participants

We conducted a nationwide online cross-sectional survey on hesitancy to undergo RAT among mainland Chinese adults uninfected with SARS-CoV-2 between April 29, 2022, and May 10, 2022. Participants were recruited through WeChat, which is a popular social media platform in China with over 1 billion monthly active users [18]. An online, self-administered, anonymous questionnaire was developed via Wenjuanxing, an online survey platform.

Eligibility criteria were as follows: (1) age of 18 years or older; (2) no previous infection with SARS-CoV-2; (3) residing in mainland China; (4) ability to complete the survey in Chinese; and (5) willingness to participate in the survey. To improve the quality of collected data, responses were deleted if (1) there were logically contradictory answers and (2) the time to complete the questionnaire was less than 2 minutes.

This survey based on a convenience sampling method [19,20] (assuming a 60% response rate) had access to respondents with diverse demographic, socioeconomic, and geographic characteristics in China. The questionnaire was comprised of closed-ended questions. Respondents were required to respond to all closed-ended questions so that there were no missing values for those questions. To avoid unmotivated, dishonest, inattentive, or duplicate responses, we tried to improve the quality of the questionnaire responses by deleting such responses. Moreover, in the process of data cleaning, we set rigorous standards for valid responses from participants.

### Ethical Considerations

The nationwide cross-sectional survey was conducted with the approval of the Ethics Committee of the School of Public Health (Shenzhen), Sun Yat-sen University (approval number: SYSU-SPH2022020). Respondents were recruited without compensation. Respondents had to provide informed consent before proceeding to the questionnaire response page in the survey.

This study was a secondary analysis of data from the survey; therefore, this study received an exemption from the Ethics Committee of the School of Public Health (Shenzhen), Sun Yat-sen University. The study data were deidentified.

### Measures

The questionnaire was adapted based on past literature on similar topics [16,21,22] and was revised by a panel of epidemiologists. A pilot study was conducted to help improve the questionnaire. All closed-ended questions had a single-answer or multiple-answer format, including “yes/no” scale, nominal and ordinal scale, and Likert scale questions. The questionnaire covered the following 3 parts: (1) sociodemographic characteristics; (2) experiences of COVID-19 restrictions and knowledge of COVID-19; and (3) attitude toward COVID-19 and its screening.

#### Sociodemographic Characteristics

The sociodemographic variables considered in this study were gender, age, ethnicity, socioeconomic status, residence, education level, marital status, age of other family members, occupation type, monthly salary during the COVID-19 pandemic, change in monthly salary during the COVID-19 pandemic, presence of chronic diseases (cardiovascular diseases, respiratory diseases, diabetes, cancers, liver diseases, and renal diseases, among others), and frailty status. Participants from 31 provinces, autonomous regions, and municipalities in mainland China were reclassified into the following 3 groups of socioeconomic status [23]: high (eastern region), medium (central region), and low (western region). The 5-item FRAIL scale (fatigue, resistance, ambulation, illness, and loss of weight) was used to identify frail persons at risk of developing disability, as well as decline in health functioning and mortality [24]. FRAIL scale scores range from 0 to 5 (0, best; 5, worst) and represent robust (0), prefrail (1-2), and frail (3-5) health statuses.

#### Experiences of COVID-19 Restrictions and Knowledge of COVID-19

Experiences of COVID-19 restrictions and knowledge of COVID-19 included experience of any NPIs, number of roommates, self-reported number of close contacts (physical contact distance of <1 meter) daily, COVID-19 vaccination status (COVID-19 vaccines include inactivated vaccines administered on a 2-dose schedule, recombinant subunit vaccines administered on a 3-dose schedule, or recombinant adenovirus type 5–vectored vaccines administered as a single dose), number of PCR tests for COVID-19 in the last month, frequency of attention to information about COVID-19, and information sources and health literacy about COVID-19. Health literacy about COVID-19 specifically refers to an individual’s ability to access, comprehend, and apply information related to COVID-19. Having adequate health literacy about COVID-19 is essential for individuals to take appropriate measures to prevent the spread of the virus, protect themselves and their loved ones, and make informed decisions about COVID-19–related health care options. Examples of health literacy about COVID-19 include understanding how the virus spreads, knowing the symptoms and when to seek medical attention, following public health guidelines, such as wearing masks and practicing physical distancing, and understanding the safety and efficacy of COVID-19 vaccines. In addition, we used a 5-point Likert scale (strongly disagree, disagree, neither agree nor disagree, agree, and strongly agree) to assess items relating to health literacy about COVID-19.

#### Attitude Toward COVID-19 and Its Screening

Questions about attitude toward COVID-19 and its screening were asked, including perceived risk of infection of COVID-19, worries about COVID-19 infection, self-assessed mental health status, perceived burden or stress during the COVID-19 pandemic, and hesitancy about RAT. Mental health disorders related to COVID-19 refer to a range of psychological and emotional conditions that have arisen as a result of the COVID-19 pandemic. We asked respondents if they experienced mental health disorders related to the pandemic in the survey questionnaire. Respondents were asked whether they were unsure or unwilling to undergo SARS-CoV-2 RAT. The question we used in the questionnaire was as follows: “When SARS-CoV-2 RAT becomes available, will you take it?” The response options were “yes,” “not sure,” and “no.” Based on the answers, respondents were classified into the following 2 groups: willing and hesitant (including the answers of “not sure” and “no”).

### Statistical Analysis

To tackle the question of the age of other family members with the multiple-answer format, the categorical variable was reclassified as follows: the age of other family members was reclassified by whether a participant had children (<6 years old) or elders (>60 years old). For other questions with the multiple-answer format, categorical variables were converted into binary dummy variables. Likert scale–type questions relating to health literacy about COVID-19 were treated as continuous variables. We calculated proportions or medians with interquartile ranges for all variables, stratified by hesitancy to undergo RAT. Chi-square tests (for proportions) and Wilcoxon rank sum tests (for medians) were used to assess statistically significant differences between the willing and hesitant groups.

Logistic regression models were fitted to the data to assess correlates of hesitancy to undergo RAT. The outcome variable was whether a participant was willing to undergo RAT (coded as 0) or was hesitant (coded as 1). In addition, the questionnaire data had a group structure for all categorical variables, including 25 groups (ie, questions or covariates) that consisted of 44 variables (ie, the sum of [the number of answer choices for each binary dummy variable − 1], the number of other categorical variables, and the number of continuous variables). Due to the multicollinearity among covariates in regression analysis, we employed a variable selection step to determine the optimal covariate sets. In this study, to control multicollinearity effectively by assessing covariates with groups of variables as opposed to independent variables and to select the optimum group of variables by inducing sparsity in the number of covariates, we used the sparse group minimax concave penalty (MCP), which has been proven to outperform other penalties, such as the group LASSO (least absolute shrinkage and selection operator) [25]. Once the optimal penalty was estimated, the final logistic regression models were refitted without the penalty to debias the shrunken estimates in the coefficients due to the penalty [26]. All statistical analyses were performed using R software version 4.2.1 (R Core Team).

## Results

### Characteristics of the Study Participants

**Figure 1:**
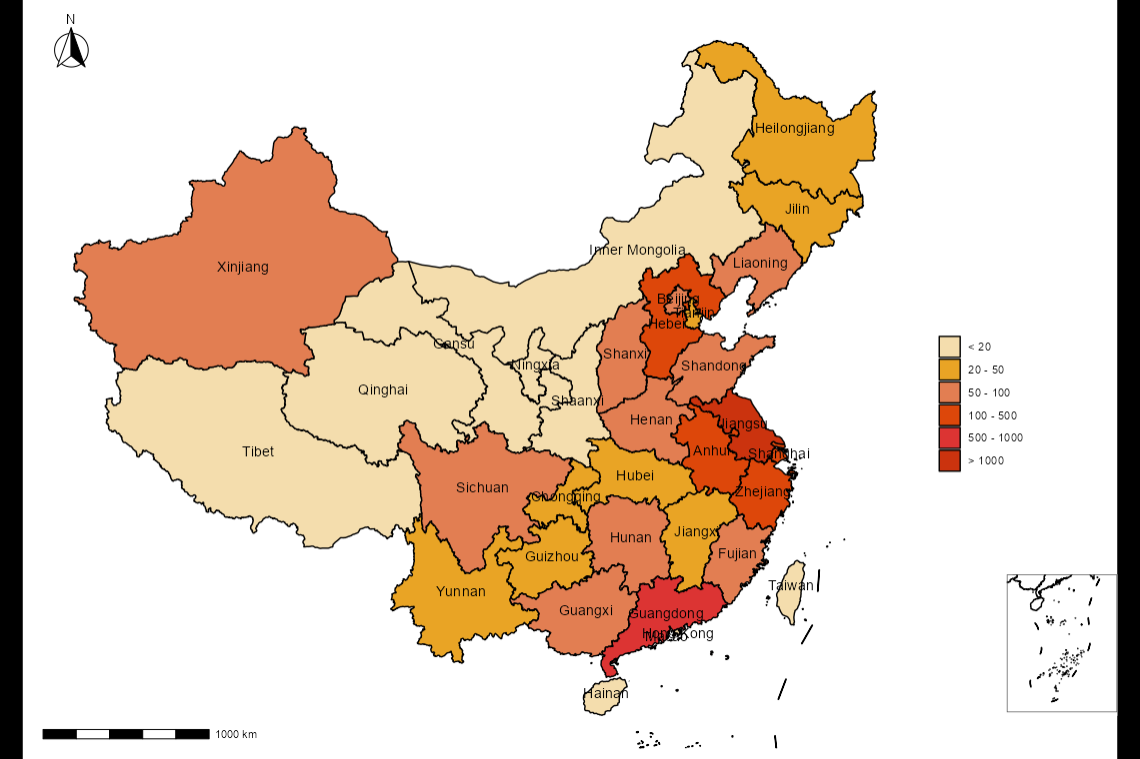
Geographical distribution of the study participants.

This survey had access to 8856 respondents with diverse demographic, socioeconomic, and geographic characteristics in China. In the process of data cleaning, we set rigorous standards for valid responses from participants. Ultimately, a total of 5388 participants (response rate of 60.84%; adults nationwide) was set as the fixed sample size. Figure 1 shows the geographical distribution (province level) of study participants in China. Among the participants, 2819 were women, and the proportions of women and men were 52.32% (2819/5388) and 47.68% (2569/5388), respectively. The median age of all participants was 32 years. In this survey, 88.33% (4759/5388) of participants were from urban areas, 80.31% (4327/5388) had an undergraduate education or above, 28.64% (1543/5388) were professional technicians, and 26.90% (1449/5388) had a monthly salary of at least 10,000 RMB (1 RMB=0.14 USD). With regard to health status, 86.06% (4637/5388) had absence of chronic diseases. Moreover 56.01% (3018/5388) had experienced any NPIs and 73.20% (3944/5388) were receiving or had completed the booster vaccination against COVID-19.

In this survey, among the 5388 participants, 687 (12.75%) expressed hesitancy to undergo RAT for COVID-19 and 4701 (87.25%) were willing to undergo RAT. The characteristics of study participants in the RAT willing and hesitant groups are shown in Tables 1-3. The 2 groups were roughly similar with respect to ethnicity, monthly salary, and COVID-19 vaccination status. Among 4701 participants in the willing group, 2529 (53.80%) were women, while among 687 participants in the hesitant group, 290 (42.21%) were women. The willing group was significantly older than the hesitant group (*P*<.001). Those in the willing group were more likely to be in the eastern region (3940/4701, 83.81% vs 479/687, 69.72%), be in urban areas (4186/4701, 89.04% vs 573/687, 83.41%), have a postgraduate education (901/4701, 19.17% vs 79/687, 11.50%), be married (2843/4701, 60.48% vs 387/687, 56.33%), and have elders in the family (2513/4701, 53.45% vs 248/687, 36.10%) than those in the hesitant group. In addition, the other variables (except ethnicity, monthly salary, and COVID-19 vaccination status) were correlated with willingness to undergo RAT.

**Table 1:** Sociodemographic characteristics of study participants by hesitancy to undergo rapid antigen testing.

Variable			Overall (N=5388)		Willing group (n=4701)		Hesitant group (n=687)		*P* value
**Gender, n (%)**									<.001
	Men	2569 (47.68)		2172 (46.20)		397 (57.79)			
	Women	2819 (52.32)		2529 (53.80)		290 (42.21)			
Age, median (IQR)			32 (26-40)		32 (26-40)		29 (23-36)		<.001
**Ethnicity, n (%)**									.64
	Han	5163 (95.82)		4507 (95.87)		656 (95.49)			
	Others	225 (4.18)		194 (4.13)		31 (4.51)			
**Socioeconomic status^a^, n (%)**									<.001
	High (eastern)	4419 (82.02)		3940 (83.81)		479 (69.72)			
	Medium (central)	654 (12.14)		487 (10.36)		167 (24.31)			
	Low (western)	315 (5.85)		274 (5.83)		41 (5.97)			
**Residence, n (%)**									<.001
	Urban	4759 (88.33)		4186 (89.04)		573 (83.41)			
	Rural	629 (11.67)		515 (10.96)		114 (16.59)			
**Education, n (%)**									<.001
	High school or below	1061 (19.69)		891 (18.95)		170 (24.75)			
	Bachelor’s degree	3347 (62.12)		2909 (61.88)		438 (63.76)			
	Master’s degree or above	980 (18.19)		901 (19.17)		79 (11.50)			
**Marital status, n (%)**									.002
	Single	1982 (36.79)		1694 (36.03)		288 (41.92)			
	Married	3230 (59.95)		2843 (60.48)		387 (56.33)			
	Divorced/widowed	176 (3.27)		164 (3.49)		12 (1.75)			
**Age of other family members, n (%)**									<.001
	Not having children (<6 years old) or elders (>60 years old)	1952 (36.23)		1640 (34.89)		312 (45.41)			
	Having children (<6 years old)	675 (12.53)		548 (11.66)		127 (18.49)			
	Having elders (>60 years old)	1919 (35.62)		1748 (37.18)		171 (24.89)			
	Having children (<6 years old) and elders (>60 years old)	842 (15.63)		765 (16.27)		77 (11.21)			
**Occupation, n (%)**									<.001
	Company employee	789 (14.64)		652 (13.87)		137 (19.94)			
	Health care professional	278 (5.16)		251 (5.34)		27 (3.93)			
	Professional technician	1543 (28.64)		1350 (28.72)		193 (28.09)			
	Student	588 (10.91)		508 (10.81)		80 (11.64)			
	Public servant	781 (14.50)		713 (15.17)		68 (9.90)			
	Self-employed	291 (5.40)		260 (5.53)		31 (4.51)			
	Teacher	264 (4.90)		211 (4.49)		53 (7.71)			
	Service worker	151 (2.80)		138 (2.94)		13 (1.89)			
	Unemployed	194 (3.60)		161 (3.42)		33 (4.80)			
	Retiree	202 (3.75)		184 (3.91)		18 (2.62)			
	Others	307 (5.70)		273 (5.81)		34 (4.95)			
**Monthly salary (RMB)^b^, n (%)**									.17
	<5000	1174 (21.79)		1030 (21.91)		144 (20.96)			
	5000-10,000	1849 (34.32)		1611 (34.27)		238 (34.64)			
	10,001-15,000	739 (13.72)		647 (13.76)		92 (13.39)			
	15,001-20,000	314 (5.83)		281 (5.98)		33 (4.80)			
	>20,000	396 (7.35)		354 (7.53)		42 (6.11)			
	No fixed salary	916 (17.00)		778 (16.55)		138 (20.09)			
**Change in monthly salary, n (%)**									<.001
	No change	2602 (48.29)		2318 (49.31)		284 (41.34)			
	Decrease by 10% or less	696 (12.92)		581 (12.36)		115 (16.74)			
	Decrease by more than 10%	1976 (36.67)		1708 (36.33)		268 (39.01)			
	Increase by 10% or less	56 (1.04)		44 (0.94)		12 (1.75)			
	Increase by more than 10%	58 (1.08)		50 (1.06)		8 (1.16)			
**Presence of chronic diseases (multiple answers), n (%)**									
	None	4637 (86.06)		4061 (86.39)		576 (83.84)		.07	
	Cardiovascular diseases	393 (7.29)		327 (6.96)		66 (9.61)		.01	
	Respiratory diseases	155 (2.88)		117 (2.49)		38 (5.53)		<.001	
	Diabetes	161 (2.99)		128 (2.72)		33 (4.80)		.003	
	Cancer	84 (1.56)		63 (1.34)		21 (3.06)		.001	
	Liver diseases	45 (0.84)		32 (0.68)		13 (1.89)		.001	
	Renal diseases	43 (0.80)		35 (0.74)		8 (1.16)		.25	
	Other diseases	133 (2.47)		126 (2.68)		7 (1.02)		.009	
**Frailty status, n (%)**									<.001
	Robust	2770 (51.41)		2458 (52.29)		312 (45.41)			
	Prefrail	2462 (45.69)		2134 (45.39)		328 (47.74)			
	Frail	156 (2.90)		109 (2.32)		47 (6.84)			

^a^The eastern region includes Beijing, Tianjin, Hebei, Liaoning, Shanghai, Jiangsu, Zhejiang, Fujian, Shandong, Guangdong, and Hainan. The central region includes Shanxi, Jilin, Heilongjiang, Anhui, Jiangxi, Henan, Hubei, and Hunan. The western region includes Sichuan, Chongqing, Guizhou, Yunnan, Tibet, Shaanxi, Gansu, Qinghai, Ningxia, Xinjiang, Inner Mongolia, and Guangxi.

^b^A currency exchange rate of 1 RMB=0.14 USD is applicable.

**Table 2:** Experiences of COVID-19 restrictions and knowledge of COVID-19 by hesitancy to undergo rapid antigen testing.

Variable			Overall (N=5388)		Willing group (n=4701)		Hesitant group (n=687)		*P* value
**Experience of any NPIs^a^, n (%)**									<.001
	No	2370 (43.99)		2155 (45.84)		215 (31.30)			
	Yes	3018 (56.01)		2546 (54.16)		472 (68.70)			
**Number of roommates, n (%)**									.02
	0	664 (12.32)		573 (12.19)		91 (13.25)			
	1	1218 (22.61)		1046 (22.25)		172 (25.04)			
	2-5	3371 (62.56)		2973 (63.24)		398 (57.93)			
	>5	135 (2.51)		109 (2.32)		26 (3.78)			
**Self-reported number of close contacts daily^b^, n (%)**									<.001
	0-5	2113 (39.22)		1813 (38.57)		300 (43.67)			
	6-10	1358 (25.20)		1166 (24.80)		192 (27.95)			
	11-20	782 (14.51)		684 (14.55)		98 (14.26)			
	21-30	313 (5.81)		288 (6.13)		25 (3.64)			
	>30	822 (15.26)		750 (15.95)		72 (10.48)			
**COVID-19 vaccination status^c^, n (%)**									.10
	Basic vaccination phase	1246 (23.13)		1066 (22.68)		180 (26.20)			
	Booster vaccination phase	3944 (73.20)		3458 (73.56)		486 (70.74)			
	No	198 (3.67)		177 (3.77)		21 (3.06)			
**Number of PCR^d^ tests for COVID-19 in the last month, n (%)**									<.001
	0-5	1155 (21.44)		855 (18.19)		300 (43.67)			
	6-10	1691 (31.38)		1504 (31.99)		187 (27.22)			
	11-20	1657 (30.75)		1528 (32.50)		129 (18.78)			
	>20	885 (16.43)		814 (17.32)		71 (10.33)			
**Frequency of attention to information about COVID-19, n (%)**									<.001
	Often	4641 (86.14)		4089 (86.98)		552 (80.35)			
	Sometimes	622 (11.54)		508 (10.81)		114 (16.59)			
	Rarely	108 (2.00)		92 (1.96)		16 (2.33)			
	Never	17 (0.32)		12 (0.26)		5 (0.73)			
**Information sources (multiple answers), n (%)**									
	Not interested in any information	17 (0.32)		12 (0.26)		5 (0.73)		.04	
	Internet media	4955 (91.96)		4359 (92.72)		596 (86.75)		<.001	
	Local authorities	3410 (63.29)		2985 (63.50)		425 (61.86)		.41	
	Traditional media	2409 (44.71)		2016 (42.88)		393 (57.21)		<.001	
	Friends or family members	2069 (38.40)		1804 (38.37)		265 (38.57)		.92	
	Others	439 (8.15)		397 (8.45)		42 (6.11)		.04	
Health literacy about COVID-19, median (IQR)			11.0 (7.0-13.0)		12.0 (8.0-13.0)		10.0 (6.0-12.0)		<.001

^a^NPI: nonpharmaceutical intervention.

^b^Close contacts refer to contacts with a physical contact distance of <1 meter.

^c^COVID-19 vaccines include inactivated vaccines administered on a 2-dose schedule, recombinant subunit vaccines administered on a 3-dose schedule, or recombinant adenovirus type 5–vectored vaccines administered as a single dose.

^d^PCR: polymerase chain reaction.

**Table 3:** Attitude toward COVID-19 and its screening by hesitancy to undergo rapid antigen testing.

Variable			Overall (N=5388)		Willing group (n=4701)		Hesitant group (n=687)		*P* value
**Perceived risk of infection of COVID-19, n (%)**									.005
	Low	1684 (31.25)		1440 (30.63)		244 (35.52)			
	Medium	2700 (50.11)		2359 (50.18)		341 (49.64)			
	High	1004 (18.63)		902 (19.19)		102 (14.85)			
**Worry about COVID-19 if infected (multiple answers), n (%)**									
	Worry about health	4098 (76.06)		3583 (76.22)		515 (74.96)		.47	
	Worry about discrimination	1955 (36.28)		1696 (36.08)		259 (37.70)		.41	
	Worry about others	682 (12.66)		634 (13.49)		48 (6.99)		<.001	
	No worry	574 (10.65)		489 (10.40)		85 (12.37)		.12	
**Self-assessed mental health status, n (%)**									<.001
	General	1822 (33.82)		1623 (34.52)		199 (28.97)			
	Good	334 (6.20)		276 (5.87)		58 (8.44)			
	Better	271 (5.03)		210 (4.47)		61 (8.88)			
	Relatively poor	2595 (48.16)		2291 (48.73)		304 (44.25)			
	Poor	366 (6.79)		301 (6.40)		65 (9.46)			
**Perceived burden and stress (multiple answers), n (%)**									
	None	242 (4.49)		217 (4.62)		25 (3.64)		.25	
	Postal and delivery services	2628 (48.78)		2295 (48.82)		333 (48.47)		.87	
	Nationwide travel restrictions	2643 (49.05)		2335 (49.67)		308 (44.83)		.02	
	Financial insecurity	2267 (42.07)		1971 (41.93)		296 (43.09)		.57	
	Medical services	1410 (26.17)		1209 (25.72)		201 (29.26)		.05	
	Mental health disorders	1999 (37.10)		1810 (38.50)		189 (27.51)		<.001	
	Burden of work	2267 (42.07)		1971 (41.93)		296 (43.09)		.57	
	Social isolation	1818 (33.74)		1599 (34.01)		219 (31.88)		.27	
	Others	164 (3.04)		156 (3.32)		8 (1.16)		.002	

### Correlates of Hesitancy to Undergo RAT

**Figure 2:**
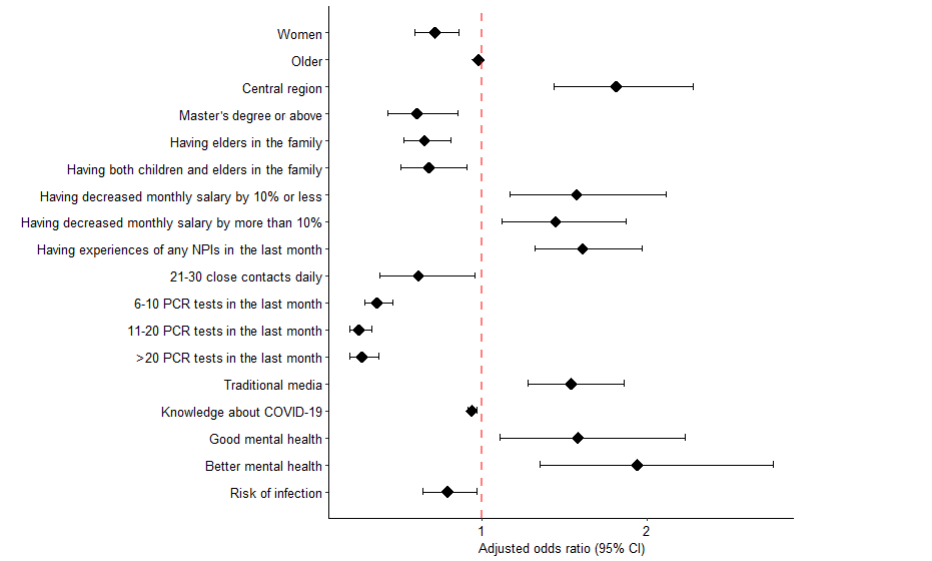
Forest plot summarizing only the significant variables that were associated with hesitancy to undergo SARS-CoV-2 rapid antigen testing. The central region includes Shanxi, Jilin, Heilongjiang, Anhui, Jiangxi, Henan, Hubei, and Hunan. Close contacts refer to contacts with a physical contact distance of <1 meter. NPI: nonpharmaceutical intervention; PCR: polymerase chain reaction.

In total, 21 groups that consisted of 40 variables selected by the MCP-penalized group regression were included in the final model. Only 13 groups were significantly associated with developing hesitancy to undergo RAT for COVID-19 (Table 4). The forest plot given in Figure 2 reveals the difference in the adjusted odds ratios (aORs) of the risk factors of hesitancy to undergo SARS-CoV-2 RAT between the willing group and hesitant group. Actually, Figure 2 shows the forest plot summarizing only the significant variables that were associated with hesitancy to undergo RAT. The complete table containing all variables left in the final model is presented in Multimedia Appendix 1.

Six sociodemographic variables were selected to be included in the model. Those who were women (aOR 0.720, 95% CI 0.599-0.864), were older (aOR 0.982, 95% CI 0.969-0.995), had a postgraduate education (aOR 0.612, 95% CI 0.435-0.858), and had children and elders in the family (aOR 0.685, 95% CI 0.510-0.911) showed willingness to undergo RAT. However, those who were from the central region (aOR 1.815, 95% CI 1.441-2.278) and had a decrease in the monthly salary by more than 10% (aOR 1.449, 95% CI 1.123-1.875) were significantly more likely to report hesitancy to undergo RAT for COVID-19 (*P*<.001 and *P*=.005, respectively). Five variables concerning experiences of COVID-19 restrictions and knowledge of COVID-19 were also selected to be included in the model. Those who had 21-30 close contacts daily (aOR 0.621, 95% CI 0.386-0.960), had >20 PCR tests for COVID-19 (aOR 0.279, 95% CI 0.205-0.377), and had more knowledge about COVID-19 (aOR 0.942, 95% CI 0.916-0.970) showed greater ease in accepting RAT. In addition, those who experienced any NPIs (aOR 1.613, 95% CI 1.322-1.973) and received COVID-19 information from traditional media (aOR 1.544, 95% CI 1.279-1.863) were significantly more likely to report hesitancy to undergo RAT for COVID-19 (both *P*<.001). Two psychological and attitudinal variables were also found to be significantly associated with developing hesitancy to undergo RAT for COVID-19. Those who had a better self-assessed mental health status (aOR 1.943, 95% CI 1.352-2.765) were significantly more likely to report hesitancy to undergo RAT for COVID-19 (*P*<.001), while those who had mental health disorders (aOR 0.795, 95% CI 0.646-0.975) showed higher wiliness to undergo RAT.

**Table 4:** Correlates of hesitancy to undergo rapid antigen testing.

Variable					aOR^a^ (95% CI)			*P* value
**Sociodemographic characteristics**								
	**Gender**							
		Men	Reference			N/A^b^		
		Women	0.720 (0.599-0.864)			<.001		
	Age			0.982 (0.969-0.995)			.006	
	**Socioeconomic status^c^**							
		High (eastern)	Reference			N/A		
		Medium (central)	1.815 (1.441-2.278)			<.001		
		Low (western)	0.787 (0.531-1.140)			.22		
	**Education**							
		High school or below	Reference			N/A		
		Bachelor’s degree	0.878 (0.699-1.107)			.27		
		Master’s degree or above	0.612 (0.435-0.858)			.005		
	**Age of other family members**							
		Not having children (<6 years old) or elders (>60 years old)	Reference			N/A		
		Having children (<6 years old)	1.149 (0.879-1.497)			.31		
		Having elders (>60 years old)	0.659 (0.530-0.817)			<.001		
		Having children (<6 years old) and elders (>60 years old)	0.685 (0.510-0.911)			.01		
	**Change in monthly salary**							
		No change	Reference			N/A		
		Decrease by 10% or less	1.577 (1.172-2.117)			.002		
		Decrease by more than 10%	1.449 (1.123-1.875)			.005		
		Increase by 10% or less	1.234 (0.554-2.575)			.59		
		Increase by more than 10%	1.546 (0.634-3.350)			.30		
**Experiences of COVID-19 restrictions and knowledge of COVID-19**								
	**Experience of any NPIs^d^**							
		No	Reference			N/A		
		Yes	1.613 (1.322-1.973)			<.001		
	**Self-reported number of close contacts daily^e^**							
		0-5	Reference			N/A		
		6-10	0.981 (0.790-1.217)			.86		
		11-20	0.989 (0.754-1.290)			.94		
		21-30	0.621 (0.386-0.960)			.04		
		>30	0.755 (0.558-1.010)			.06		
	**Number of PCR^f^ tests for COVID-19 in the last month**							
		0-5	Reference			N/A		
		6-10	0.370 (0.296-0.463)			<.001		
		11-20	0.262 (0.203-0.336)			<.001		
		>20	0.279 (0.205-0.377)			<.001		
	**Information sources (multiple answers)**							
		Not interested in any information	Reference			N/A		
		Internet media	0.780 (0.577-1.064)			.11		
		Local authorities	0.852 (0.699-1.040)			.11		
		Traditional media	1.544 (1.279-1.863)			<.001		
		Friends or family members	1.073 (0.881-1.304)			.48		
		Others	0.863 (0.597-1.219)			.42		
		Health literacy about COVID-19	0.942 (0.916-0.970)			<.001		
**Attitude toward COVID-19 and its screening**								
	**Self-assessed mental health status**							
		General	Reference			N/A		
		Good	1.583 (1.109-2.232)			.01		
		Better	1.943 (1.352-2.765)			<.001		
		Relatively poor	1.114 (0.897-1.387)			.33		
		Poor	1.337 (0.930-1.904)			.11		
	**Perceived burden and stress (multiple answers)**							
		None	Reference			N/A		
		Postal and delivery services	1.058 (0.876-1.277)			.56		
		Nationwide travel restrictions	0.947 (0.779-1.150)			.58		
		Financial insecurity	0.875 (0.716-1.069)			.19		
		Medical services	1.104 (0.898-1.353)			.34		
		Mental health disorders	0.795 (0.646-0.975)			.03		
		Burden of work	1.142 (0.935-1.392)			.19		
		Social isolation	1.149 (0.934-1.413)			.19		
		Others	0.614 (0.268-1.221)			.20		

^a^aOR: adjusted odds ratio.

^b^N/A: not applicable.

^c^The eastern region includes Beijing, Tianjin, Hebei, Liaoning, Shanghai, Jiangsu, Zhejiang, Fujian, Shandong, Guangdong, and Hainan. The central region includes Shanxi, Jilin, Heilongjiang, Anhui, Jiangxi, Henan, Hubei, and Hunan. The western region includes Sichuan, Chongqing, Guizhou, Yunnan, Tibet, Shaanxi, Gansu, Qinghai, Ningxia, Xinjiang, Inner Mongolia, and Guangxi.

^d^NPI: nonpharmaceutical intervention.

^e^Close contacts refer to contacts with a physical contact distance of <1 meter.

^f^PCR: polymerase chain reaction.

## Discussion

In this nationwide cross-sectional survey, we found that 12.75% (687/5388) of the participants were hesitant to undergo RAT for COVID-19, while 87.25% (4701/5388) were willing to undergo RAT. Several characteristics were found to be associated with hesitancy to undergo RAT, which could help identify subgroups to improve the awareness and acceptance of RAT.

Hesitancy about COVID-19 in China was low but may compromise the scale-up of RAT. A cross-sectional survey involving residents of Greece and Cyprus found that 79% (196/248) reported willingness to self-test in fighting the COVID-19 pandemic [27]. In another representative survey of the Greek population conducted in the first half of August 2021, two-thirds of responders characterized COVID-19 self-tests as unreliable and two-fifths believed they were dangerous [28]. An Indonesia study found that, if rapid COVID-19 antigen self-tests were available, 62.70% (395/630) would use them when necessary [29]. In addition to positive attitudes toward COVID-19 vaccination [30], this relatively high percentage of willingness to undergo RAT in China might reflect public trust in the government, and health authorities in China spared no effort to offer accessible health care services to increase safety and the perceptions of safety for the public. As the COVID-19 pandemic continues and in the postpandemic era, it would be important to monitor and promote public willingness toward RAT further in order to identify infected individuals in the early phase and keep COVID-19 incidence low, as well as improve the coverage of routine RAT for COVID-19.

Hesitancy to undergo RAT was found to be associated with being male, which is similar to the epidemiologic characteristic in the Greek population [28]. In addition, younger individuals reported low willingness to undergo RAT compared to their older counterparts. This association was also found in a representative survey of 17 countries on COVID-19 vaccine hesitancy [31]. There could be some possible explanations. Younger individuals may perceive themselves as being at lower risk of experiencing a severe illness associated with COVID-19, as the disease tends to affect older people more severely. This perception of lower risk could lead them to believe that RAT is unnecessary. In addition, younger individuals may have lower incomes or be more financially insecure, and the cost of RAT may be a barrier for them. They may also be more concerned about missing work or school if they test positive and need to isolate. Moreover, we found that socioeconomic status and education might play a role in determining hesitancy to undergo RAT. Areas further to the east in China have a better socioeconomic status and higher education level, and a better socioeconomic status and higher education level have been previously found to be associated with better health literacy and lower vaccine hesitancy [32,33]. This might have resulted in a situation where those who were from the central region and had a high school education or lower were significantly more likely to report hesitancy to undergo RAT, similar to vaccination for COVID-19. A change in the monthly salary during the COVID-19 lockdown was much more likely to be associated with hesitancy to undergo SARS-CoV-2 RAT compared with the current monthly salary. This could indicate that those who experience job loss, reduced income, and increased debt during the COVID-19 pandemic should receive much more attention to improve the awareness and acceptance of SARS-CoV-2 RAT. The pandemic has also highlighted existing social inequalities, with communities, who experienced heavier financial burden, being disproportionately affected by the virus. A study also found that individuals with lower salary were more likely to have higher similar mistrust about vaccines [33]. In addition, we found that not having children or elders in the family was correlated with hesitancy to undergo RAT. This may be due to the desire to protect children and elders in the family and prevent ﻿chains of intrahousehold transmission. Those having elders or having both children and elders in the family showed willingness to undergo RAT.

We identified 5 variables concerning experiences of COVID-19 restrictions and knowledge of COVID-19, which were significantly associated with hesitancy to undergo RAT. Those who experienced any NPIs and had 0-5 close contacts daily were more likely to report hesitancy. A prior study has found that more social contacts and large households increased the risk of SARS-CoV-2 infection [34,35]. The finding for the experience of any NPIs or the self-reported number of close contacts daily might be because the subgroup believed there was a low risk of SARS-CoV-2 infection and had stronger faith in the health condition, compared with those who did not experience NPIs for COVID-19 or had more close contacts in daily life. Moreover, those who had 0-5 PCR tests for COVID-19 in the last month were more likely to report hesitancy. PCR testing remains the gold standard in all countries, with the majority also employing it for diagnostic and surveillance purposes [36]. Fewer PCR tests might deepen hesitancy to undergo RAT. Furthermore, those who received COVID-19 information from traditional media were more likely to report hesitancy. Over the ﻿course of fighting the COVID-19 pandemic, health-related information and knowledge have played large roles in shaping vaccine hesitancy [37]. Increased likelihood of vaccine acceptance was seen among those who obtained COVID-19 information from digital media or nonhealth-related sources like influencers [38], and this likely was related to hesitancy to undergo RAT among participants in our survey. Traditional media like ﻿newspapers are not prone to misinformation, but do not have the same tremendous and popular health information available as other information sources like internet media. Moreover, inadequate knowledge has been found to be associated with vaccine hesitancy [39], which might be in line with our finding that those who had less knowledge about COVID-19 were significantly more likely to report hesitancy to undergo RAT.

We identified 2 psychological and attitudinal variables that were significant predictors of hesitancy to undergo RAT. Those who had good or better self-assessed mental health and did not perceive a high risk for SARS-CoV-2 infection were more likely to report hesitancy, which might be attributable to the belief that one ought to undergo RAT if they are at high risk for SARS-CoV-2 infection. This finding suggests that the government should offer appropriate recommendations on RAT, with the aim of identifying infected individuals in the early phase and keeping COVID-19 incidence low to tackle outbreaks in the future and especially to safeguard the shift from a pandemic phase to an endemic phase of the COVID-19 global response.

To reliably scale up RAT further for case management or self-isolation at the individual level and for large-scale screening and emergency responses at the population level, the government should offer more accurate recommendations on RAT to the target population. First, the public should be provided with the test as widely and freely as possible, which could be a strategy to improve the availability of RAT and the willingness to undergo RAT without additional financial cost for the public. Second, trust about RAT should be enhanced, including enough knowledge about COVID-19 and clear instructions about RAT via multiple media. Furthermore, the public should be provided with complete guidelines after undergoing RAT. For example, accessible PCR testing could be used to validate the results of RAT owing to the risk of false-positive results. If positive results are confirmed, guidelines about case management or self-isolation should be put in place.

SARS-CoV-2 has infected up to 900 million people as of January 11, 2023, in Mainland China after lifting of the COVID-19 policy [40]. The emergence of new variants (the Omicron sublineage XBB [41]) of the virus has also raised concerns about their potential to evade existing vaccines and treatments, highlighting the importance of ongoing surveillance and research. Given the reopening of society, vaccines; medication; and public health measures, such as masking, social distancing, and screening remain important tools in controlling the spread of the virus. Furthermore, by fully using the lessons learned from the mass screening (including timely scale-up of RAT) during the COVID-19 pandemic, this study could help to develop more resilient and prepared mass screening strategies, and could inform future responses to other novel infectious diseases.

In this study, we used group regression with an MCP penalty to discern variables that significantly shaped hesitancy to undergo RAT. It was found that several sociodemographic variables, variables concerning experiences of COVID-19 restrictions and knowledge of COVID-19, and psychological and attitudinal variables were associated with hesitancy to undergo RAT for COVID-19. Notably, variables, such as residence, marital status, and occupation type, were not selected for inclusion in the final model despite statistically significant differences between the study groups, which suggests that group regression with an MCP penalty was successful in not only selecting significant variables but also encouraging model sparsity. Some limitations of this study are worth acknowledging. In this survey, 88.33% of participants were from urban areas and 80.31% had an undergraduate education or above. Thus, it is very likely that individuals from rural areas and those with lower education are underrepresented. Individuals with SARS-CoV-2 infection were not included in the analysis. In addition, the sample size in this study was small compared to the large population size of China, which is another limitation of the study. Therefore, our findings should be interpreted and generalized with caution. Moreover, regarding hesitancy to undergo RAT, a single question was asked to assess hesitancy. Despite its simplicity and efficiency, more multidimensional items should be adopted to assess hesitancy in future studies. In addition, the general bias for a cross-sectional design could not be dismissed, which limited our capacity to statistically discern causal relationships. Furthermore, given the reopening of society, the aim was no longer completely relevant, considering the lifting of global and Chinese preventive and control measures for COVID-19. However, the real-world evidence in this study could inform future responses to other novel infectious diseases.

In conclusion, our study found a low but potentially problematic level of hesitancy to undergo RAT. Although hesitancy to undergo SARS-CoV-2 RAT in China was low, it may influence the scale-up of RAT. Special efforts should be made to improve the awareness and acceptance of RAT among men, younger adults, individuals with a lower education or salary, families without children and elders, and individuals who access COVID-19 information via traditional media. Given the gradual reopening of society, this study could inform our responses to future novel infectious diseases and help to develop more resilient and prepared mass screening strategies (including timely scale-up of RAT).

## Appendix

Multimedia Appendix 1 All variables left in the final model for correlates of hesitancy to undergo rapid antigen testing.

## Abbreviations

aOR: adjusted odds ratio
MCP: minimax concave penalty
NPI: nonpharmaceutical intervention
PCR: polymerase chain reaction
RAT: rapid antigen testing

## Data Availability

The deidentified data set used in this study and the R codes are publicly available and can be accessed through the following GitHub repository: [42].
