# Supplementary material for: Hesitancy to Undergo SARS-CoV-2 Rapid Antigen Testing in China: Nationwide Cross-sectional Study
Source: JMIR Public Health Surveill. 2023 May 1;9:e43555. doi: 10.2196/43555 (PMC10154020; doi:10.2196/43555)
Supplement: Multimedia Appendix 1 [file publichealth_v9i1e43555_app1.docx]

**Multimedia Appendix 1.** All variables left in the final model for correlates of hesitancy to undergo rapid antigen testing.

| Variables | ﻿Adjusted odds Ratio (95% CI) | P-value |
| --- | --- | --- |
| **Sociodemographic variables** |  |  |
| ﻿Gender |  |  |
| Men | ﻿Ref. |  |
| Women | 0.720 (0.599-0.864) | *< .001* |
| Age | 0.982 (0.969-0.995) | *.006* |
| ﻿Ethnicity |  |  |
| ﻿Han | Ref. |  |
| ﻿Others | 0.823 (0.522-1.259) | *.39* |
| ﻿Socioeconomic status1 |  |  |
| ﻿High (Eastern) | Ref. |  |
| ﻿Medium (Central) | 1.815 (1.441-2.278) | *< .001* |
| ﻿Low (Western) | 0.787 (0.531-1.140) | *.22* |
| ﻿Residence |  |  |
| ﻿Urban | Ref. |  |
| ﻿Rural | 1.229 (0.950-1.579) | *.11* |
| ﻿Education |  |  |
| ﻿High school and below | Ref. |  |
| ﻿Bachelor | 0.878 (0.699-1.107) | *.27* |
| ﻿Master and above | 0.612 (0.435-0.858) | *.005* |
| ﻿Marital status |  |  |
| ﻿Single | Ref. |  |
| ﻿Married | 1.130 (0.866-1.478) | *.37* |
| ﻿Divorced/Widowed | 0.754 (0.367-1.432) | *.41* |
| Age of other family members |  |  |
| Not having children (< 6 years old) or elders (> 60 years old) | Ref. |  |
| Having children (< 6 years old) | 1.149 (0.879-1.497) | *.31* |
| Having elders (> 60 years old) | 0.659 (0.530-0.817) | *< .001* |
| Having children (< 6 years old) and elders (> 60 years old) | 0.685 (0.510-0.911) | *.01* |
| ﻿Monthly salary |  |  |
| < 5000 RMB | Ref. |  |
| 5000-10000 RMB | 1.220 (0.954-1.565) | *.12* |
| 10001-15000 RMB | 1.354 (0.982-1.863) | *.06* |
| 15001-20000 RMB | 1.500 (0.950-2.319) | *.07* |
| > 20000 RMB | 1.400 (0.921-2.097) | *.11* |
| No fixed salary | 1.187 (0.848-1.665) | *.32* |
| Change in monthly salary |  |  |
| No change | Ref. |  |
| Decrease by 10% or less | 1.577 (1.172-2.117) | *.002* |
| Decrease by more than 10% | 1.449 (1.123-1.875) | *.005* |
| Increase by 10% or less | 1.234 (0.554-2.575) | *.59* |
| Increase by more than 10% | 1.546 (0.634-3.350) | *.30* |
| Presence of chronic diseases (MA) |  |  |
| None | Ref. |  |
| Cardiovascular diseases | 1.264 (0.894-1.767) | *.18* |
| Respiratory diseases | 0.916 (0.555-1.479) |  |
| Diabetes | 0.861 (0.519-1.395) | *.55* |
| Cancers | 1.541 (0.809-2.840) | *.18* |
| Liver diseases | 1.119 (0.501-2.378) | *.78* |
| Renal diseases | 0.572 (0.221-1.341) | *.22* |
| Other diseases | 0.466 (0.192-0.959) | *.06* |
| Frailty status |  |  |
| Robust | Ref. |  |
| Pre-frail | 1.069 (0.890-1.285) | *.47* |
| Frail | 1.254 (0.799-1.944) | *.32* |
| **Experiences of COVID-19 restrictions and knowledge of COVID-19** |  |  |
| Experience of any NPIs2 |  |  |
| No | Ref. |  |
| Yes | 1.613 (1.322-1.973) | *< .001* |
| Self-reported number of close contacts daily3 |  |  |
| 0-5 | Ref. |  |
| 6-10 | 0.981 (0.790-1.217) | *.86* |
| 11-20 | 0.989 (0.754-1.290) | *.94* |
| 21-30 | 0.621 (0.386-0.960) | *.04* |
| > 30 | 0.755 (0.558-1.010) | *.06* |
| Number of PCR testing for COVID-19 in last month4 |  |  |
| 0-5 | Ref. |  |
| 6-10 | 0.370 (0.296-0.463) | *< .001* |
| 11-20 | 0.262 (0.203-0.336) | *< .001* |
| > 20 | 0.279 (0.205-0.377) | *< .001* |
| Frequency of attention to information about COVID-19 |  |  |
| ﻿Often | Ref. |  |
| ﻿Sometimes | 1.015 (0.784-1.306) | *.91* |
| ﻿Rarely | 0.658 (0.346-1.180) | *.18* |
| Never | 1.258 (0.344-4.106) | *.71* |
| Information sources (MA) |  |  |
| Not interested in any information | Ref. |  |
| Internet media | 0.780 (0.577-1.064) | *.11* |
| Local authorities | 0.852 (0.699-1.040) | *.11* |
| Traditional media | 1.544 (1.279-1.863) | *< .001* |
| Friends or family members | 1.073 (0.881-1.304) | *.48* |
| Others | 0.863 (0.597-1.219) | *.42* |
| Health literacy about COVID-19 | 0.942 (0.916-0.970) | *< .001* |
| **Attitude towards COVID-19 and its screening** |  |  |
| Worry about COVID-19 if infected (MA) |  |  |
| No worry | Ref. |  |
| Worry about health | 0.851 (0.686-1.058) | *.14* |
| Worry about discrimination | 0.903 (0.747-1.089) | *.29* |
| Worry about others | 0.789 (0.559-1.093) | *.17* |
| Self-assessed mental health status |  |  |
| General | Ref. |  |
| Good | 1.583 (1.109-2.232) | *.01* |
| Better | 1.943 (1.352-2.765) | *< .001* |
| Relatively poor | 1.114 (0.897-1.387) | *.33* |
| Poor | 1.337 (0.930-1.904) | *.11* |
| Perceived burden and stress (MA) |  |  |
| None | Ref. |  |
| Postal and delivery services | 1.058 (0.876-1.277) | *.56* |
| Nationwide travel restrictions | 0.947 (0.779-1.150) | *.58* |
| Financial insecurity | 0.875 (0.716-1.069) | *.19* |
| Medical services | 1.104 (0.898-1.353) | *.34* |
| Mental health disorders | 0.795 (0.646-0.975) | *.03* |
| Burden of work | 1.142 (0.935-1.392) | *.19* |
| Social isolation | 1.149 (0.934-1.413) | *.19* |
| Others | 0.614 (0.268-1.221) | *.20* |

Note: 1 Eastern region includes Beijing, Tianjin, Hebei, Liaoning, Shanghai, Jiangsu, Zhejiang, Fujian, Shandong, Guangdong, Hainan. Central region includes Shanxi, Jilin, Heilongjiang, Anhui, Jiangxi, Henan, Hubei, Hunan. Western region includes Sichuan, Chongqing, Guizhou, Yunnan, Tibet, Shaanxi, Gansu, Qinghai, Ningxia, Xinjiang, Inner Mongolia, Guangxi. 2 NPIs refer to nonpharmaceutical interventions. 3 Close contacts refer to contacts that physical contacting distance is less than one meter. 4 PCR refers to polymerase chain reaction.

MA refers to Multiple-answer question.
